# A pilot study to assess the feasibility of endoscopic placement of a transurethral urinary balloon catheter in male sheep cadavers

**DOI:** 10.1186/s13028-019-0487-8

**Published:** 2019-11-04

**Authors:** Marlene Sickinger, Reto Neiger, Axel Wehrend

**Affiliations:** 1 Clinic for Obstetrics, Gynecology and Andrology of Large and Small Animals, Justus-Liebig-University of Giessen, 35392 Giessen, Germany; 2 Present Address: Clinic for Ruminants, Internal Medicine and Surgery, Justus-Liebig-University of Giessen, 35392 Giessen, Germany; 3 Clinic for Companion Animals Internal Medicine, Justus-Liebig-University of Giessen, 35392 Giessen, Germany; 4 Present Address: Hofheim Animal Hospital, 65719 Hofheim, Germany

**Keywords:** Endoscopy, Procedure, Small ruminants, Urolithiasis

## Abstract

Surgery of obstructive urolithiasis in small ruminants is often unsatisfactory due to postoperative development of strictures. The present study aimed to establish an endoscopic technique for the placement of a transurethral urinary catheter into the bladder of rams. This catheter was used as a removable stent-like drainage. The procedure was performed in three sheep rams that were euthanized and placed for surgery in 45° Trendelenburg position. In one ram, cystotomy was performed via right paramedian laparotomy. A 3 mm flexible fiberscope was introduced into the urinary bladder and advanced via urethra to the tip of the penis. Placing a guide wire through the endoscopic working channel into the urethra enabled the retrograde insertion of a transurethral urinary catheter into the bladder. In two rams, retrograde insertion of a fiberscope was performed. Again, a guidewire was used to insert a balloon catheter into the bladder. Paramedian right laparotomy was performed to ascertain the correct position of the balloon. Both techniques, antero- and retrograde endoscopy, were possible and could be successfully performed. Mucous membranes and urinary microliths were easily observed. Repeated advancing of the endoscope or the catheter resulted in marked damage of the mucous membranes. The patency of the urethra may be restored by means of endoscopic placement of a transurethral catheter in male small ruminants. The applicability and clinical outcome of this procedure as well as the effects on stricture formation should be further examined with controlled clinical studies.

## Findings

**Fig. 1:**
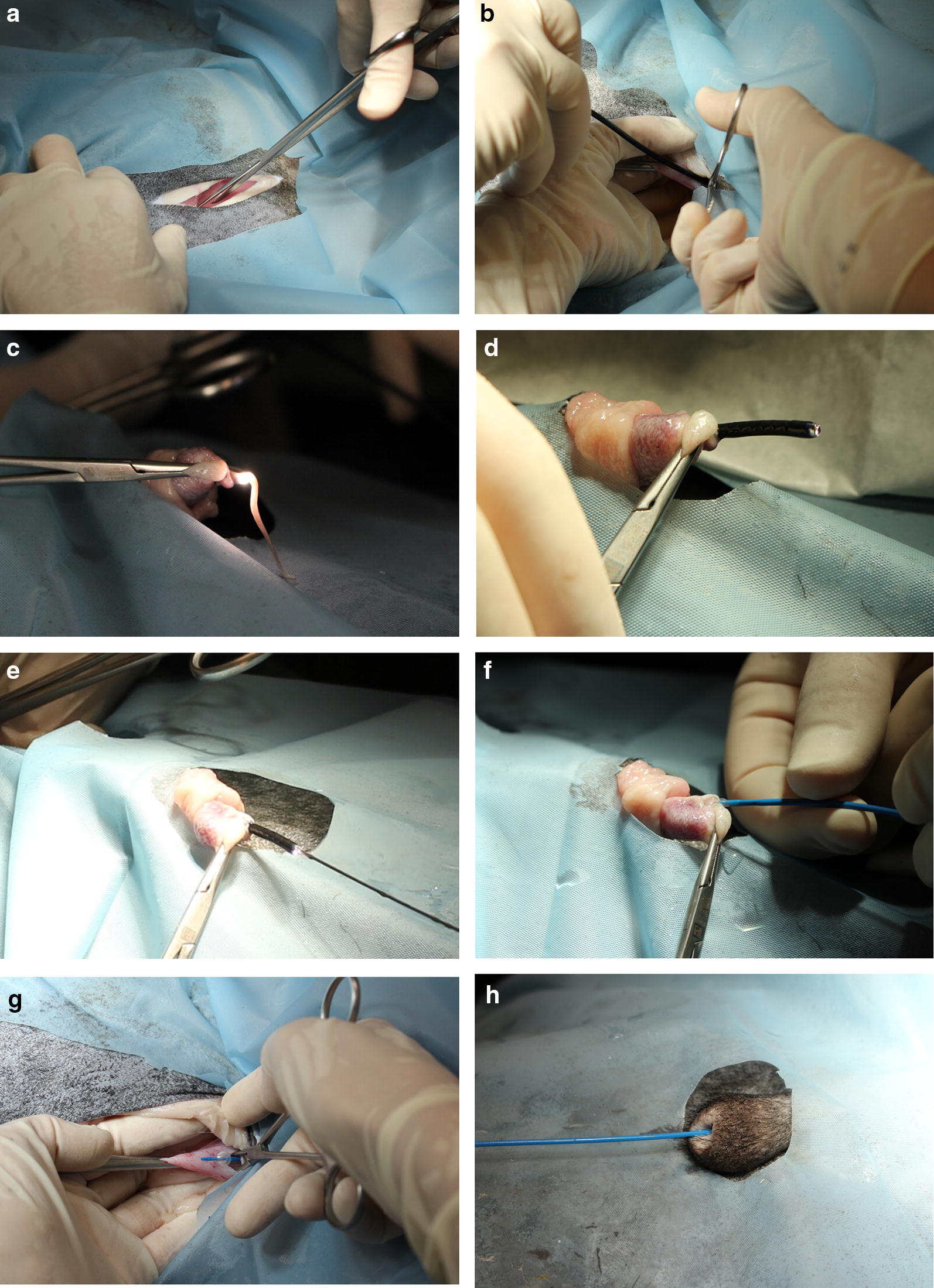
Anterograde urethroscopy and retrograde placement of a transurethral balloon-catheter into the urinary bladder. **a** Paramedian right laparotomy. **b** Insertion of the fiberscope into the urinary bladder via stab incision. **c** Fiberscope at the tip of the penis. **d** Protrusion after removing the urethral process. **e** Introduction of a guide wire into the working channel of the fiberscope with subsequent. **f** Introduction of the balloon-catheter via guide wire into the urinary bladder (**g**). **h** Catheter in situ

**Fig. 2:**
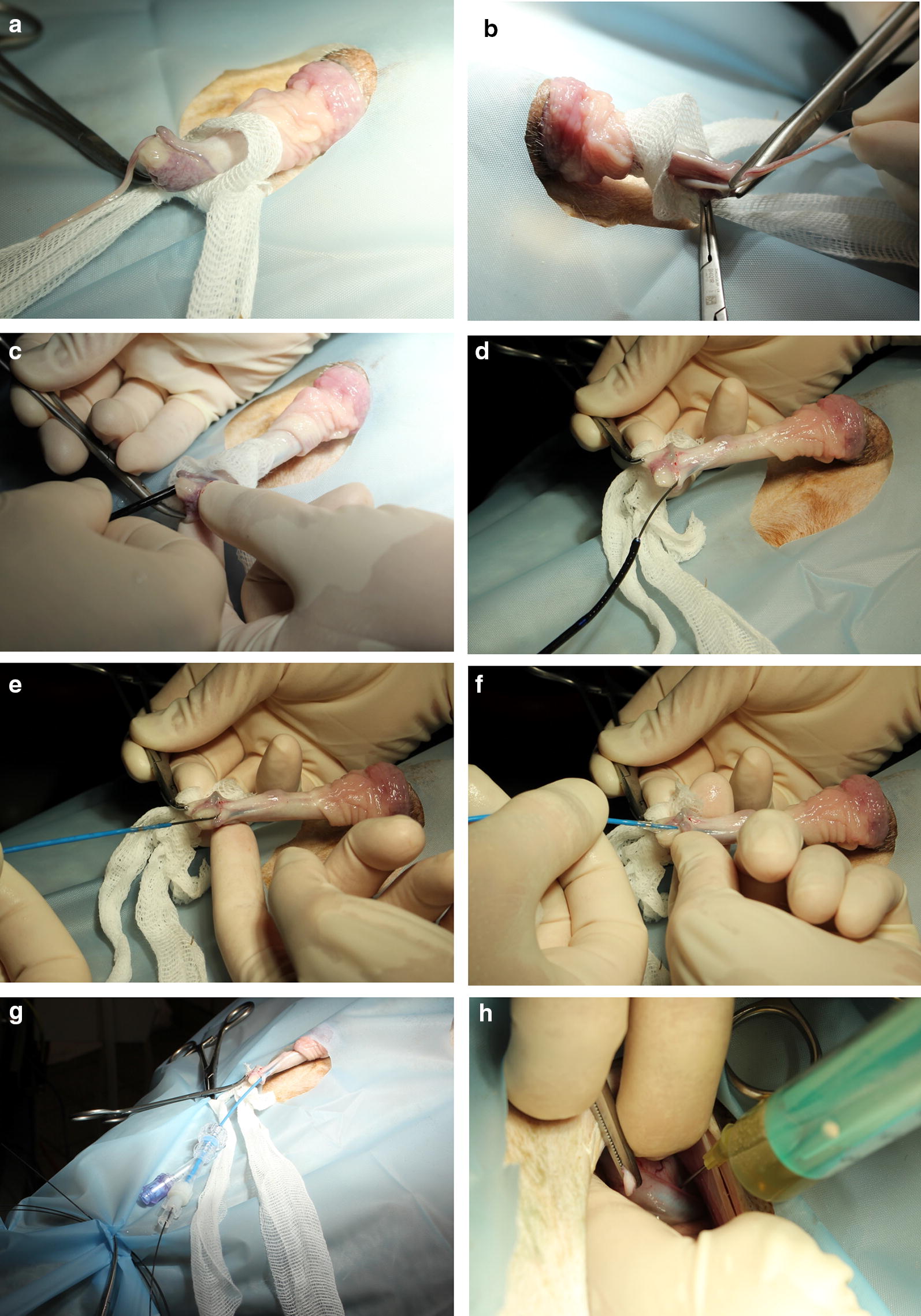
Retrograde urethroscopy and placement of the balloon-catheter. **a** Protrusion of the penis out of the prepuce. **b** Amputation of the urethral process. **c** Introduction of the fiberscope into the urethra. **d** Placing of guide wire into the urinary bladder under endoscopic control. **e–g** Introduction of the urethral catheter via guide wire into the urethra and urinary bladder. **h** Diagnostic laparotomy to ascertain the correct position of the balloon-catheter in the trigone region

Partial or complete obstruction of the urethra with potentially live threatening complications such as marked hyperkalaemia, hydronephrosis and peritonitis are potential problems of urolithiasis in small ruminants [1, 2]. Predisposing factors for the development of uroliths, such as species, breed predispositions and castration as well as dietary risk factors have been identified [3–5]. If untreated, complete urethral obstruction often results in a fatal outcome and animals are commonly presented in an emergency setting [2, 6]. Although many surgical techniques for relieve of obstructive urolithiasis have been established [7], long-term success rates are still unsatisfactory [8]. One of the main problems in small ruminants with urolithiasis is a stricture formation of the urethra after surgical intervention [9]. This complication commonly results in a relapse due to newly formed uroliths that again obliterate the urethra. The aim of the present study was to establish an endoscopic method that enables the placement of a transurethral urinary catheter into the urinary bladder. This then serves as continuous urine drainage and should prevent stricture formation as it works like a removable urethral stent (Figs. 1, 2).

The endoscopic technique was performed in three 6-months-old male intact sheep rams of different breeds (Coburger, Coburger-crossbred and Black Milksheep-crossbred). The median body weight was 38.1 kg with a range from 34.8 to 41.8 kg. Animals were euthanized with sodium-pentobarbital (500 mg/mL; Release^®^, WDT, Garbsen, Germany) immediately before starting the procedure. The study was performed in accordance with national legislation and with the approval of the local ethics committee of the Justus-Liebig-University of Giessen, Germany (Number of compliancy: 600_M/2016). The animals were prepared for surgery and positioned in dorsal recumbency in a 45°-Trendelenburg position (head lower than body).

In one ram, a paramedian laparotomy was performed and the urinary bladder was advanced. After removal of urine via cystocentesis, a flexible fiberscope (3 mm diameter, 1 m long; Storz, Tuttlingen, Germany) was advanced via cystotomy into the urethra. The mucosal surface of the urethra was evaluated and urinary microliths were identified. The endoscope was then pushed anterograde through the urethra. The urethral process was amputated to enable the endoscope to exit the urethra. A guide wire was then placed through the working channel of the endoscope. After removing the fiberscope, a ureteral occlusion balloon catheter (CH 5, balloon 1 mL, length 75 cm; Urotech, Achenmühle, Germany) was placed retrograde over the guide wire into the urinary bladder. The balloon was blocked with 1 mL NaCl. Aspiration and flushing of the urinary bladder through this catheter was easy.

In two rams, placed also in a 45° Trendelenburg position, direct retrograde cystoscopy was achieved with the same endoscope after amputating the urethral process. Passage of the urethral recess at the arcus ischiadicus during retrograde urethroscopy could only be accomplished using a Seldinger technique with the guide wire in the working channel as guide. The guide wire was left in place and after removal of the fiberscope, a CH 5 occlusion balloon catheter was placed retrograde into the bladder. Flushing and aspiration urine was equally possible.

The complete procedure could be performed in all three animals with anterograde or retrograde insertion of the fiberscope through the entire urethra. Repeated insertion and protrusion of the catheter or the fiberscope, respectively, led to marked damage of the mucosal surface of the urethra and to an enhancement of resistance when pushing the fiberscope or catheter forward. An in-depth evaluation of the urinary bladder with the small diameter fiberscope in a retrograde fashion was not possible because of insufficient illumination.

To our knowledge, this is the first study performing an endoscopy-guided catheterization of the extremely long and narrow urethra of small ruminants. In contrast to other studies, no urethrotomy was performed and a balloon catheter was placed directly into the urinary bladder. This represents a modification and combination of existing surgical techniques [10–16]. The advantage of our technique is that the placement of the urinary catheter helps to allow passage of urine, and to keep the urethra open in a fashion similar to a removable stent. Surgical techniques always have the risk of stricture formation and special procedures like buccal mucosal graft urethroplasty for reversal of an urethrostomy [9] are not suitable for most clinicians. Clinical cases will now need to be treated with this method to evaluate it in a practical setting and assess the development of strictures. In contrast to other studies [9, 17], this study indicates that this technique will be of high impact for future surgical interventions in rams with obstructive urolithiasis.

Endoscopic removal of uroliths from the urethra of male ruminants has been reported previously. The authors used endoscopy-guided laser lithotripsy in 15 goats [10] and one bull [18] with restored patency of the urethra. Due to necessary equipment and cost associated with endoscopy and lithotripsy, this method seems more suitable for companion animals [19] than being acceptable for livestock animals. However, sheep and goats are being kept more and more as companion animals, thus higher medical costs are probably accepted by the owners.

A possible drawback of our technique in anaesthetized rams could be that the long, narrow and curved urethra might not be easily stretched to accommodate the fiberscope. Using epidural anaesthesia might allow to advance the penis and to stretch the sigmoid flexure even if the animal is in dorsal recumbency [20].

In summary, endoscopic placement of a transurethral balloon-catheter into the urinary bladder is possible in an anterograde and retrograde fashion. This should decrease stricture formation due to a stent-like device into the urethra. Although this procedure is promising, further research in anaesthetized rams with urethral obstruction is necessary and must be the aim of future studies.

## Data Availability

The datasets used and/or analysed during the current study are available from the corresponding author on reasonable request.
